# Leveraging a disulfidptosis/ferroptosis-based signature to predict the prognosis of lung adenocarcinoma

**DOI:** 10.1186/s12935-023-03125-z

**Published:** 2023-11-09

**Authors:** Xiaoqing Ma, Zilin Deng, Zhen Li, Ting Ma, Guiqing Li, Cuijia Zhang, Wentao Zhang, Jin Chang

**Affiliations:** 1 Department of Radiation Oncology, The Second Affiliated Hospital of Shandong First Medical University, Taian, Shandong China; 2 Shandong First Medical University, Jinan, Shandong China

**Keywords:** Disulfidptosis, Ferroptosis, LUAD, Prognosis, Signature

## Abstract

**Background:**

Disulfidptosis and Ferroptosis are two novel forms of cell death. Although their mechanisms differ, research has shown that there is a relationship between the two. Investigating the connection between these two forms of cell death can further deepen our understanding of the development and progression of cancer, and provide better prediction models for accurate prognosis.

**Methods:**

In this study, RNA sequencing (RNA-seq) data, clinical data, single nucleotide polymorphism (SNP) data, and single-cell sequencing data were obtained from public databases. We used weighted gene co-expression network analysis (WGCNA) and unsupervised clustering to identify new Disulfidptosis/Ferroptosis-Related Genes (DFRG), and constructed a LASSO COX prognosis model that was externally validated. To further explore this novel signature, pathway and function analysis was performed, and differences in gene mutation frequency between high- and low-risk groups were studied. Importantly, we also conducted research on immune checkpoint, immune cell infiltration levels and immune resistance indicators, in addition to analyzing real clinical immunotherapy data.

**Results:**

We have identified four optimal disulfidptosis/ferroptosis-related genes (ODFRGs) that are differentially expressed and associated with the prognosis of Lung Adenocarcinoma (LUAD). These genes include GMPR, MCFD2, MRPL13, and SALL2. Based on these ODFRGs, we constructed a robust prognostic model in this study, and the high-risk group showed significantly lower overall survival (OS) compared to the low-risk group. Furthermore, this model can also predict the immunotherapy outcomes of LUAD patients to some extent.

**Supplementary Information:**

The online version contains supplementary material available at 10.1186/s12935-023-03125-z.

## Introduction

Lung cancer is one of the main causes of cancer death worldwide. According to statistics from the World Health Organization, more than 1.8 million people die from lung cancer each year [1]. Among them, lung adenocarcinoma is the most common type, accounting for 40–50% of cases [2]. The early prognosis model study has shown that the treatment effect of lung cancer is affected by the death mode, and thus, the role of these death modes needs to be studied more deeply. The treatment of lung adenocarcinoma is challenging, with poor prognosis. Current treatments have many side effects and risks, and their impacts on survival and long-term effectiveness are still uncertain. Therefore, researchers are actively searching for new treatment methods and strategies, such as immunotherapy, gene therapy, targeted therapy, interventional therapy, and exploring molecular biomarkers of lung adenocarcinoma prognosis. These studies aim to bring new breakthroughs and progress in the treatment and prognosis of lung adenocarcinoma.

Disulfidptosis and Ferroptosis are two regulatory forms of cell death. Disulfidptosis is a novel form of cell death recently discovered in the field of biology. When the expression level of the Solute Carrier Family 7 Member 11 (SLC7A11) gene is high in cells and encounters glucose deprivation, disulfidptosis is triggered [3]. Its defining characteristic is the formation of compounds containing disulfide bonds inside the cell, which accumulate on the cell's actin cytoskeleton, leading to cytoskeleton collapse and eventual cell death. SLC7A11 has a dual regulatory role in cell death/survival and redox homeostasis. High expression of SLC7A11 promotes cell death due to rapid depletion of NADPH and accumulation of toxic cystine. Disulfidptosis is a distinct mechanism of cell death that involves disruption of actin filaments, unlike ferroptosis. The regulation of disulfidptosis involves the formation and rupture of disulfide bonds, as well as the involvement of proteins such as NCKAP1 and signaling pathways related to redox and cellular metabolism. The significance of disulfidptosis lies in its potential as a target for cancer therapy, especially in cancer cells with high expression of SLC7A11. Inhibition of glucose transporter proteins (GLUT) has shown therapeutic efficacy against SLC7A11-high expressing cancer cells by inducing disulfidptosis. Various methods, including glucose deprivation experiments, cystine accumulation analysis, disulfide detection, observation of actin collapse, and cell death assays, have been used to characterize and study disulfidptosis [4, 5]. Overall, understanding the regulatory mechanisms and significance of disulfidptosis contributes to a deeper exploration of cellular homeostasis and provides potential strategies for targeted cancer therapy. As for ferroptosis, it is a special form of iron-dependent cell death that primarily occurs through lipid peroxidation and cell membrane damage [6]. In cancer therapy, ferroptosis plays a crucial role. Tumor cells often contain higher levels of iron and have a stronger dependence on it compared to normal cells [7]. Therefore, by inducing ferroptosis, it is possible to selectively kill cancer cells while causing minimal damage to non-malignant cells. Compared to other forms of cell death, the molecular mechanisms of ferroptosis are more unique. It involves a series of specific molecular processes and pathways [6], such as iron accumulation, lipid peroxidation, oxidative stress, and cell membrane disruption. Studies have found that ferroptosis can be induced through various pathways, including endogenous and exogenous pathways [8–10]. The endogenous pathway is initiated by inhibiting cell membrane transport proteins (such as cystine/glutamate transporter) or activating iron transport proteins (such as transferrin and lactoferrin). The exogenous pathway, on the other hand, activates by blocking intracellular antioxidant enzymes (such as glutathione peroxidase 4, GPX4). GPX4 is an important regulatory factor in ferroptosis, inhibiting its occurrence by reducing the formation of phospholipid hydroperoxides [11]. Ferroptosis is not only associated with the sensitivity of cancer cells to anticancer drugs but also closely related to tumor initiation and development. Studies have confirmed that overexpression of GPX4 and decreased expression of SLC7A11 are associated with treatment resistance in certain types of cancer [8]. Therefore, inducing ferroptosis holds potential as a therapeutic strategy for tumors. Depending on the specific tumor type, iron content, gene expression levels, and mutations, appropriate drugs can be selected to promote ferroptosis and achieve more effective treatment outcomes [10].

Although Disulfidptosis and Ferroptosis are two independent forms of cell death, they share common regulatory factors. The expression of the important regulatory gene SLC7A11 in Disulfidptosis can affect the iron ion content in cells [12–15]. Therefore, in this study, we innovatively link Disulfidptosis and Ferroptosis, and screened out Disulfidptosis/Ferroptosis-related genes through public databases. These genes can be used as markers for Disulfidptosis and Ferroptosis. We have constructed a prognosis model related to DFRGs to predict the prognosis and immune score of LUAD patients. Based on this study, we hope to provide help for personalized treatment of patients.

## Material and methods

### Data resources

This study obtained RNA-seq data and clinical data of lung adenocarcinoma patients from the TCGA database (https://portal.gdc.cancer.gov/) and the GEO database (https://www.ncbi.nlm.nih.gov/geo/, ID: GSE68465 GSE72094). Single-cell sequencing data (ID:GSE131907) and single nucleotide variation data were also obtained from the TCGA database. In addition, we obtained 512 ferroptosis-related genes and 17 disulfidptosis-related genes(DRGs) from the FerrDb database (http://www.zhounan.org/ferrdb/current/) and literature.

### Identification of genes related to disulfidptosis and ferroptosis

In this study, the RNA-seq and clinical data were preprocessed to remove missing values. Gene Set Variation Analysis (GSVA) was performed using the "GSVA" R package to obtain the enrichment scores of disulfidptosis and ferroptosis on all samples of GSE68465. Based on the GSVA results, Weighted Gene Co-expression Network Analysis (WGCNA) was conducted to identify DFRGs. The "PickSoftThreshold" function was employed to automatically select a soft threshold and different power values were used to conduct unscaled hierarchical clustering on modules. The corresponding dissimilarity matrix (1-TOM) and topological overlap matrix (TOM) were obtained. Co-expression modules were subjected to Pearson correlation analysis based on GSVA scores, and the module with the highest correlation with disulfidptosis and ferroptosis was chosen as the DFRGs identified through WGCNA.

Based on the results of R package "ConsensusClusterPlus" and GSVA, consensus clustering analysis was performed on samples related to disulfidptosis in GSE68465. The clustering variable (K) was increased from 2 to 10, and the best K value was found, which provided the highest intra-group correlation and the lowest inter-group correlation. The DRGs related clusters were analyzed using Kaplan–Meier (KM) analysis through the R packages "survival" and "survminer" to compare the differences of OS. In addition, the differential expression of 17 DRGs between the two clusters was analyzed. Then, the R package "DeSeq2" was used to perform differential analysis on the clustered genes (|log2FC|≥ 1 and FDR < 0.05) to obtain new DFRGs. Subsequently, immune scores were obtained using TIMER, ssGSEA, Cibersort, and Estimate algorithms to explore the relationship between the DRGs related clusters and the immune system.

Subsequently, unsupervised clustering analysis was performed on the training set based on the ferroptosis gene set obtained from the FerrDb database. The ferroptosis-related clusters were then analyzed for survival, clinical, and immune-related information. The intersection of the genes selected by the three methods mentioned above results in the DFRGs.

### Development and validation of the DFRGs prognostic model

Perform Univariate Cox analysis on DFRGs, select DFRGs that are statistically significant (P < 0.05) and related to survival. Using the R packages "randomForestSRC", "glmnet", "xgboost", "gbm", "xgboost", and "survival", evaluate the weight calculation average related to DFRGs survival based on five machine learning algorithms: decision trees, random forests, LASSO, gradient boosting decision trees (GBDT), and extreme gradient boosting (XGBoost), and rank them. Following that, the top ten genes were chosen based on their survival weights. The lasso cox model was constructed using the R packages "glmnet" and "survival". Initially, the ten-fold cross-validation technique was applied using the "cv.glmnet" function to determine the optimal penalty coefficient (λ) for model fitting and subsequent analysis and prediction. Subsequently, the "coef" function was utilized to extract DRGs that were associated with non-zero coefficients. The risk score was calculated by summing the product of the coefficients and expression levels of these DRGs. Use the median of the training set's risk score as the cutoff value to divide all samples into high-risk and low-risk groups. Perform KM analysis on the high-risk and low-risk groups, and evaluate the model's accuracy through ROC (receiver operating characteristic curve) analysis using the "timeROC" R package. In addition, we display the differences between the high-risk and low-risk groups in terms of gender, age, and clinical stage using a heatmap to explore the relationship between risk scores and clinical information, and investigate the expression of genes used in constructing the model and their relationship with clinical information.

### Enrichment analysis related to signaling pathway and function

Based on the GSEA (Gene Set Enrichment Analysis) and GSVA (Gene Set Variation Analysis) algorithms, we conducted a comprehensive analysis of pathway and functional relationships in relation to risk subtypes. GSEA was performed using the GSEA software (version 4.2.3), with gene sets sourced from the KEGG database. GO enrichment analysis was conducted at three levels: MF (Molecular Function), CC (Cellular Component), and BP (Biological Process). In addition, we also analyzed the correlation between risk scores and tumor-related pathways as well as pathways associated with disulfidptosis and ferroptosis. Important signaling pathways in tumors include Hippo, Wnt, MAPK, PI3K/AKT, TGF-β, NF-kB, Notch, AMPK, JAK-STAT, PD-1/PD-L1, mTOR, Ras, TNF, HIF-1, and ErbB. Disulfidptosis/ferroptosis-related pathways and functions include actin cytoskeleton, glucose starvation, lipid homeostasis, tricarboxylic acid cycle, iron metabolism, P53 signaling pathway, glutathione peroxidase activity, and pentose phosphate pathway.

### Analysis of immune cell infiltration and immune score

The tumor microenvironment refers to the complex network of cells, molecules, and signaling pathways that interact with tumor cells. It has a significant impact on tumor growth, invasion, metastasis, and treatment response. In this study, we obtained evaluation results of immune cell infiltration abundance from various algorithms in the TIMER2.0 (http://timer.cistrome.org/) database, including TIMER, CIBERSORT, CIBERSORT-ABS, QUANTISEQ, MCPCOUNTER, XCELL, and EPIC. Moreover, single sample gene set enrichment analysis (ssGSEA) was conducted on LUAD samples to analyze immune cell infiltration abundance using R packages "GSVA" and "GSEABase." The ESTIMATE algorithm was used to evaluate the relationship between tumor purity and subtype, including ESTIMATE Score, Immune Score, and Stromal Score. In addition, we assessed the expression of immune checkpoints and differences in risk between risk subgroups in LUAD samples.

### Analysis of single-cell sequencing data

This study collected single-cell sequencing data from 15 LUAD samples in the GEO database. We used the Seurat R package in R language to analyze the sequencing data. Specifically, we first used the "CreateSeuratObject" function to perform preliminary screening of the original data and retained high-quality cells. Then, we used the "PercentageFeatureSet" function to calculate the percentage of mitochondrial genes in each cell, with the selection criteria being the expression of genes in at least 3 cells and the percentage of ribosomal genes below 20%. We normalized the data using the "LogNormalize" method in the "NormalizeData" function. Next, we identified highly variable genes using the "FindVariableFeature" function, performed PCA dimensionality reduction using the "RunPCA" function, and identified important principal components through the "jackstraw" function. Finally, we used the t-SNE algorithm to select the top 20 principal components for cell clustering analysis and used the "FindAllMarkers" function to annotate cell clusters and calculate differential genes for each cluster. This experiment combined single-cell sequencing technology and the "Seurat" R package in R language to explore the expression patterns of individual cells in tumor tissues, revealing the heterogeneity of tumor cells.

### Analysis of risk subtypes and gene mutations

Single-nucleotide polymorphism (SNP) data in TCGA refers to information on single-nucleotide polymorphisms in the genomic DNA of cancer cells. SNPs refer to variation that occurs at a single nucleotide (A, C, G, or T) position that may be related to the occurrence and development of cancer. TCGA SNP data provides genotype information for millions of SNPs, which can be used to study the relationship between SNPs and cancer, helping to gain a deeper understanding of the genetic basis of cancer. We obtained SNP data for LUAD from the TCGA database. The MAFTOOL software was used to display the top-ranked mutated genes in the high-risk and low-risk groups, as well as their mutation types and frequencies, and to assess the correlation between mutation counts and risk score.

### Analysis of the expression level of ODFRGs

We used RNA-seq data from normal lung samples and lung adenocarcinoma samples in the TCGA database. Subsequently, we analyzed the expression of ODFRGs (including GMPR, MCFD2, MRPL13 and SALL2) in tumor and normal samples. Furthermore, to demonstrate the reliability of the sequencing data, we also obtained the immunohistochemical results of ODFRGs in lung adenocarcinoma tissues from The Human Protein Atlas (HPA).

### Immunohistochemistry

Five Lung adenocarcinoma tissue chips were purchased from Shanghai Outdo Biotech Company (Shanghai, China).The paraffin removal process: After overnight baking in a 65 °C oven, the tissue chips were deparaffinized in xylene II solution (each for 10 min), followed by a series of ethanol gradients (100%, 95%, 80%, 70% for 2 min each), and washed three times with PBS (5 min each time). Endogenous peroxidase blocking: Remove excess water from the slides and place them in a humidified box. Add 3% hydrogen peroxide and leave at room temperature for 10 min, followed by three washes with PBS (5 min each time). Antigen retrieval: Add sodium citrate antigen retrieval solution to a pressure cooker and place the slides inside. Once the pressure cooker reaches maximum pressure, set the timer for 2.5 min. After turning off the power, let it cool to room temperature. Wash the slides three times with PBS (5 min each time). Antibody incubation: Remove excess water from the slides and place them in a humidified box. Incubate with primary antibody reagent (1:100) at 35 °C for 1 h, followed by three washes with PBS (5 min each time). Remove excess water from the slides and place them in a humidified box. Incubate with the secondary antibody of goat anti-mouse/rabbit IgG conjugated with enzyme for 30 min, followed by three washes with PBS (5 min each time). DAB staining: Prepare DAB working solution by mixing DAB buffer solution with 4u DAB concentrate per milliliter. Apply the evenly mixed DAB working solution onto each slide and observe the staining intensity under a light microscope. Rinse with distilled water to stop the staining. Counterstaining: Place the slides in a staining dish containing hematoxylin for 2 min, followed by a 1-min rinse with distilled water. Then, immerse the slides in hydrochloric acid ethanol for 2 s, followed by a 1-min rinse with distilled water. Finally, place the slides in ammonia for 10 s and rinse again with distilled water for 1 min. Dehydration: Dehydrate the slides sequentially in ethanol gradients (70%, 85%, 95% for 2 min each), and then immerse them in xylene I and II solutions for 2 min each. Place the slides in a fume hood for 2 h for ventilation. Mounting: Apply neutral adhesive onto the slides and cover them with coverslips.

Result interpretation: Semi-quantitative scoring was performed based on the staining intensity and percentage of positive cells. Under a microscope, two pathologists independently and blindly determined the immunohistochemical staining results. No staining, weak positivity (pale yellow particles), positivity (brownish-yellow particles), and strong positivity (dark brown particles) were scored as 0, 1, 2, and 3, respectively. Based on the percentage of positive stained cells relative to the total number of cells, 0% was assigned a score of 0, 1% to 25% was assigned a score of 1, 26% to 50% was assigned a score of 2, 51% to 75% was assigned a score of 3, and 75% was assigned a score of 4. The final expression score of ODFRGs for each sample was calculated by multiplying the staining intensity by the percentage score of positive cells. A score of ≤ 6 was classified as low expression, and a score of > 6 was classified as high expression. For each slide, five random high-power field views at 400 × magnification were selected, and the staining intensity and percentage of positive cells were scored for each region, with the average value being the final score result.

### Quantitative real time polymerase chain reaction (qRT-PCR)

Normal lung epithelial cells BEAS-2B and three human lung adenocarcinoma cell lines, A549, H1299, PC9, were obtained from the Central Laboratory of Shandong First Medical University Affiliated Provincial Hospital. PCR ARRAY was purchased from Shanghai Audoo Biological Technology Company (Shanghai, China). Total RNA was extracted using TRIzol reagent (Invitrogen, USA). Complementary DNA (cDNA) was synthesized using the PrimeScript RT kit (Takara).

### Western blotting

Cells were lysed in cold RIPA buffer. An equal quantity of protein was then subjected to SDS-PAGE and subsequently transferred to a PVDF membrane. The membrane was blocked with nonfat dry milk containing TBST for 1 h. The primary antibody (a universal primary antibody used for western blot and IHC) was diluted as per the manufacturer's instructions and incubated overnight at 4 °C. Following washing with TBST, the secondary antibody was added and incubated for 1 h at room temperature. After washing the membrane, it was developed using an enhanced chemiluminescence (ECL) chromogenic solution.

### CCK-8 assay

Cell proliferation was assessed using the CCK-8 assay. A549 and PC9 cells were seeded in 96-well plates and cultured for 0, 24, 48, and 72 h. Afterward, they were incubated with the CCK-8 solution in the dark for 1 h. The absorbance values were then measured at 450 nm using a microplate reader.

### Colony formation assay

A549 and PC9 cells were cultured in 6-well tissue culture plates for one week until cell colonies formed. The resulting cell colonies were fixed with 0.5% polyformaldehyde (Servicebio, Beijing, China) for 25 min and stained with 2.5% methylene violet dye for 15 min. Following washing, the cell colonies were recorded and counted.

### Wound healing assay

For the wound-healing assay, 24-well plates were utilized to seed cells. Cells were scratched perpendicular to the previously marked line using a sterile tip. Cell migration was measured at time points of 0 and 24 h after imaging the scratch wounds with a light microscope.

### Transwell assay

We performed Transwell assays using 24-well transwell chambers to evaluate the invasiveness of A549 and PC9 cell lines. Cells were seeded in the upper chambers with or without Matrigel in serum-free culture medium. The lower chambers were filled with 600 μl of culture medium containing 10% serum. After 24 h, the cells were fixed and stained.

### Statistical analysis

In this study, we used various testing methods in statistical and bioinformatics analyses, including Wilcoxon rank-sum test, Pearson's chi-squared test, t-test, and logarithmic transformation test. The Wilcoxon rank-sum test was used to compare differences between two sample groups, while Kruskal–Wallis test was used to analyze differences among sample groups with more than two samples. We set the threshold of statistical significance at p < 0.05. Data analysis was conducted using R exclusively.

## Results

### Identification of disulfidptosis/ferroptosis-related genes

**Fig. 1:**
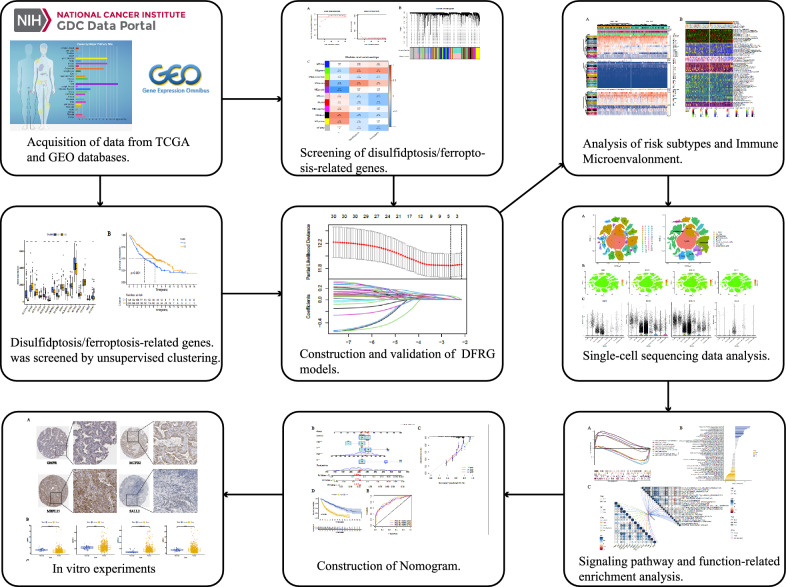
Workflow of the study. This figure shows the construction process and subsequent analysis of the DFRG model

**Fig. 2:**
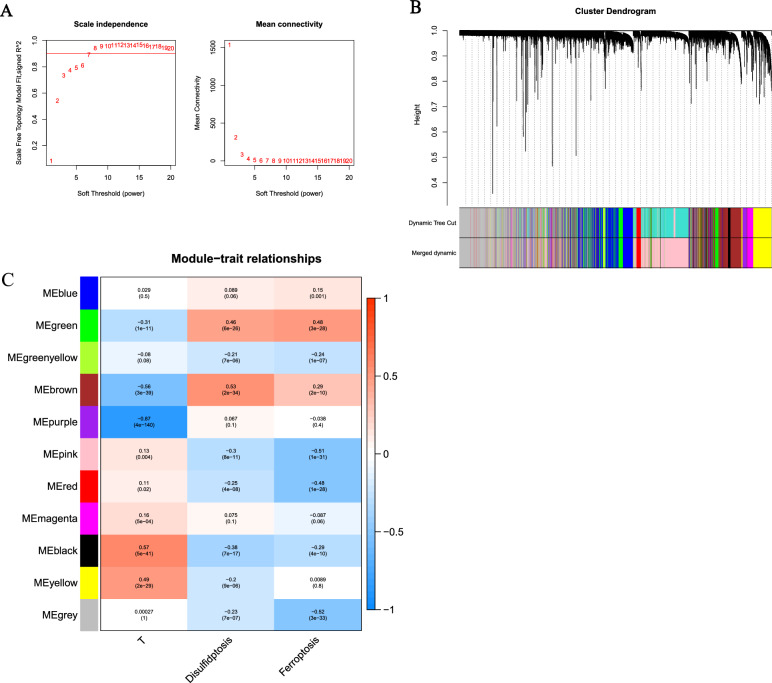
Discovery of prognostic DFRGs by WGCNA. **A** Displays the distribution and trends of the scale-free topology model fit and mean connectivity with soft threshold. **B** Shows the clustering of genes among different modules by the dynamic tree cut and merged dynamic method. The gray modules depict unclassified genes. **C** Depicts the average correlation between multiple modules and tumor development, disulfidptosis and ferroptosis. The cell color indicates the strength of correlation, while the P-value for the correlation test appears in parentheses

The specific process of this study is shown in Fig. 1. First, we used WGCNA to analyze the RNA-seq data of the training set. The "pickSoftThreshold" function in the WGCNA R package was used to automatically select a soft threshold of 7 (Fig. 2A). Multiple gene modules were identified using a dynamic cut method and all modules were further clustered using the "mergeCloseModules" function to obtain the final module (Fig. 2B). We used Pearson correlation analysis to identify the most relevant module, which was called "green" and contained a total of 970 genes related to disulfidptosis and ferroptosis (Fig. 2C).

**Fig. 3:**
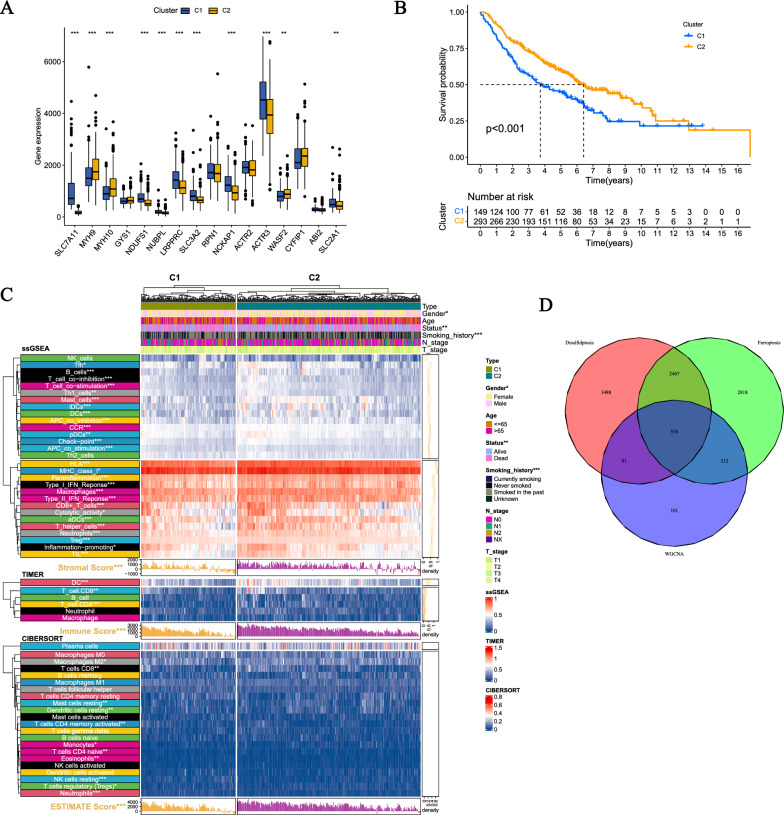
Consensus clustering for screening disulfidptosis-related genes. **A** Difference in expression of disulfidptosis-related genes between two clusters. **B** Kaplan–Meier curve for disulfidptosis-related clusters. **C** Differences in clinical data and abundance of immune cell infiltration in DRG-related clusters. (D) Using algorithms such as WGCNA and unsupervised clustering, we identified a total of 576 DFRGs

Based on DRGs, we performed unsupervised clustering of the samples from GSE68465. The cluster value (K) was evaluated for 2–10 results. The results showed that the intra-group relationships were the strongest and the cluster stability was the highest when K = 2 (Additional file 1: Fig. S1A). In addition, to further study the clusters related to disulfidptosis, we analyzed the expression of DRGs, clinical data, and immune cell infiltration levels, including ssGSEA, CIBERSORT, TIMER, and ESTIMATE. The results showed that almost all DRGs had statistically significant differences between two clusters (p < 0.05) (Fig. 3A), and the Kaplan–Meier (KM) curve showed statistically significant differences in OS of samples from cluster1 and cluster2 (Fig. 3B). Comprehensive heatmaps were constructed to analyze the clinical data and immune scores (Fig. 3C), which showed differences between the two clusters in terms of gender, smoking history, and immune cell infiltration abundance. Differential analysis of clusters related to DRGs identified 4612 new DRGs (|logFC|≥ 1, p < 0.05).

The selection of new FRGs was also obtained through unsupervised clustering, and the stability was best when K = 2 (Additional file 1: Fig. S1B). There were differences in the survival of the two clusters related to ferroptosis (Additional file 1: Fig. S1C). A total of 6074 new FRGs (|logFC|≥ 1, p < 0.05) were identified through differential analysis. Finally, a total of 576 DFRGs were screened by combining the three rounds of screening (Fig. 3D).

### Development and validation of DFRGs signature

**Fig. 4:**
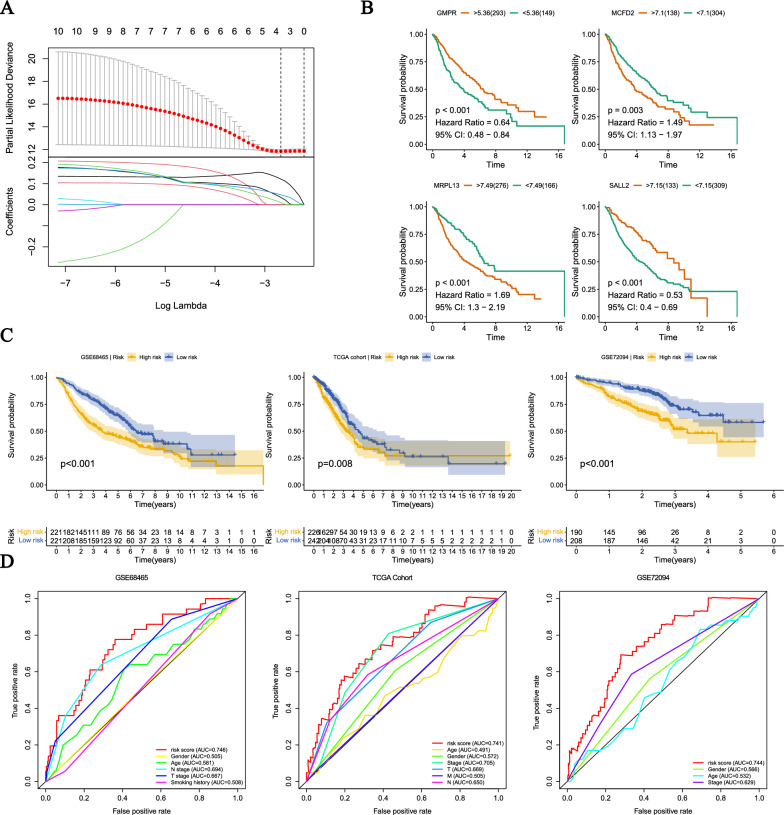
Screening of ODFRGs and construction of models. **A** Choose the best value of λ through LASSO regression and configure the LASSO coefficients. **B** There is a significant difference in survival between high and low expression groups of ODFRGs (namely GMPR, MCFD2, MRPL13, SALL2). **C** Survival differences between high-risk and low-risk groups in the training set and test set. The table below shows the number of patients still alive in each year. **D** The ROC curves showed the predictive efficiency of risk scores and clinical characteristics

To screen survival-related DFRGs, we performed univariate Cox analysis (Additional file 1: Fig. S1D). Based on machine learning algorithms, we evaluated the weights associated with survival-related DFRGs and constructed LASSO Cox models using the top 10 genes (Additional file 2: Table S1) (Fig. 4A). Next, we demonstrated the KM curves for the gene high-expression and low-expression groups in the construction of the model(Fig. 4B). The high-expression groups of GMPR and SALL2 showed higher OS rates compared to the low-expression groups, indicating their protective role. On the other hand, the low-expression groups of MCFD2 and MRPL13 showed higher OS rates compared to the high-expression groups, suggesting their association with increased risk. These analyses all exhibited statistically significant differences. The risk score calculation was as follows:

$$ {\text{Risk Score}}\, = \,\left( { - 0.{1928 }*{\text{ GMPR exp}}.} \right)\, + \,\left( {0.{2762 }*{\text{ MCFD2 exp}}.} \right)\, + \,\left( { - 0.{1568 }*{\text{ SALL2 exp}}.} \right)\, + \,\left( {0.0{837 }*{\text{ MRPL13 exp}}.} \right) $$

We selected the median value of the risk score of the training set samples as the cutoff value and divided the LUAD patients into high- and low-risk groups. The KM curves showed that the OS of patients in the high-risk group was significantly lower than that of those in the low-risk group in the training set (GSE68465) and validation set (TCGA cohort, GSE72094) (Fig. 4C). The ROC curves showed that the AUC values of the risk scores for GSE68465, TCGA cohort, and GSE72094 were 0.746, 0.741, and 0.744(Fig. 4D), respectively, indicating that the signature is highly stable.Through univariate and multivariate Cox analysis, we found that age (HR = 1.039, 95% confidence interval (CI) = 1.023–1.056, p < 0.001), T stage (HR = 1.430, 95% CI = 1.124–1.818, p = 0.004), N stage (HR = 2.096, 95% CI = 1.731–2.537, p < 0.001), and risk score (HR = 2.161, 95% CI = 1.337–3.491, p = 0.002) were independent prognostic factors (Additional file 1: Fig. S1E, F).

### Clinical analysis of signature associated with DFRGs

**Fig. 5:**
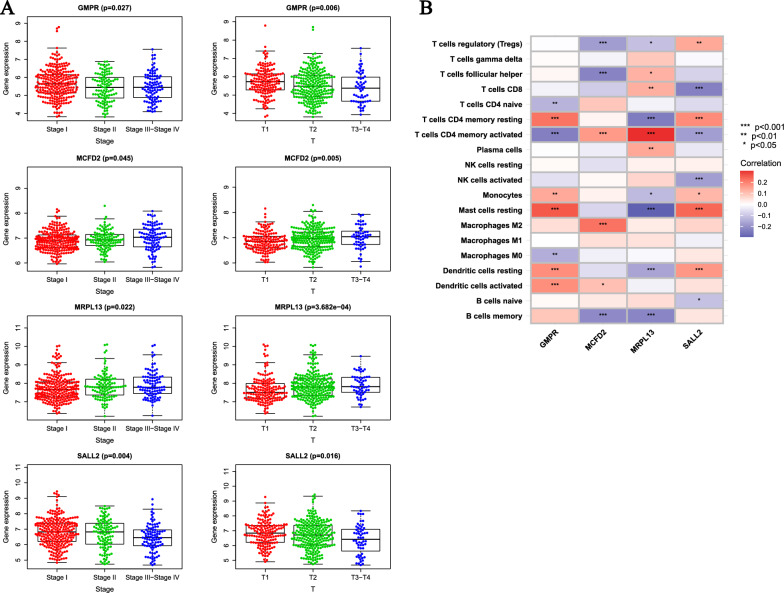
Clinical analysis and correlation analysis of immune cell infiltration for DFRGs. **A** The relationship between gene expression of GMPR, MCFD2, MRPL13, and SALL2 and tumor stage and T stage in LUAD patients. **B** Correlation analysis of expression levels of GMPR, MCFD2, MRPL13, SALL2 and abundance of immune cell infiltration (p < 0.05; p < 0.01; **p < 0.001)

**Fig. 6:**
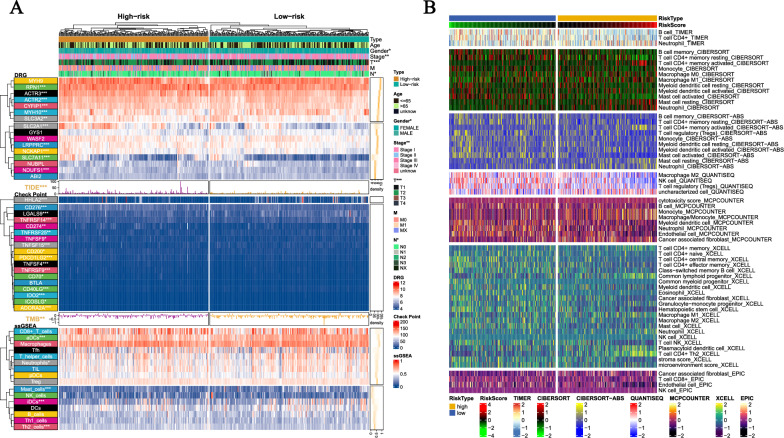
Clinical and immune analysis related to risk subgroups. **A** Differences in clinical data, DRGs, TMB, TIDE scores, and ssGSEA results between high and low-risk groups. TIDE scores and TMB are presented in the form of a bar chart and density plot respectively. **B** The differences in the abundance of immune cell infiltration algorithms in TIMER2.0 database including TIMER, CIBERSORT, CIBERSORT-ABS, MCPCOUNTER, XCELL, and EPIC between the high-risk and low-risk groups (the heatmap shows the results with statistical differences)

We used Kruskal–Wallis test to analyze the relationship between ODFRG (GMPR, MCFD2, MRPL13, SALL2) and clinical data. The results showed that there were differences in ODFRG expression levels between different clinical stages and T stages. When the expression levels of GMPR and SALL2 were high, the stage and T stage were generally higher, while MCFD2 and MRPL13 had the opposite trend (Fig. 5A). There was a correlation between GMPR, MCFD2, MRPL13, SALL2 and immune cell infiltration levels (Fig. 5B). In addition, there were differences in gender, stage, T stage, N stage between the high-risk group and the low-risk group (Fig. 6A).

### Immunological analysis of DRG signature

To verify the association between risk subtypes and disulfidptosis, the expression levels of DRGs in the high and low-risk groups were analyzed. The results showed statistical differences (p < 0.05) in the expression levels of RPN1, ACTR3, ACTR2, CYFIP1, MYH10, SLC3A2, SLC2A1, LRPPRC, NCKAP1, SLC7A11, and NDUFS1 between the high and low-risk groups. Subsequent immune checkpoint analysis showed differences in CD274 (PDL1), PDCD1LG2 (PDL2) and others between the high and low-risk groups. Furthermore, TIDE and TMB also showed differences between the high and low-risk groups. The analysis of immune cell infiltration abundance includes multiple algorithms in ssGSEA and the TIME2.0 database. The results of ssGSEA (Fig. 6A) showed that the infiltration levels of aDCs, Neutrophils, Mast cells, iDCs, and Th2 cells differed significantly (p < 0.05) between the high and low-risk groups. The results of TIMER data (Fig. 6B), including immune cell infiltration algorithms such as TIMER, CIBERSORT, CIBERSORT-ABS, QUANTISEQ, MCPCOUNTER, XCELL, and EPIC, also showed significant differences (p < 0.05) between the high and low-risk groups.

### Overview of the scRNA-Seq data generated from LUAD

**Fig. 7:**
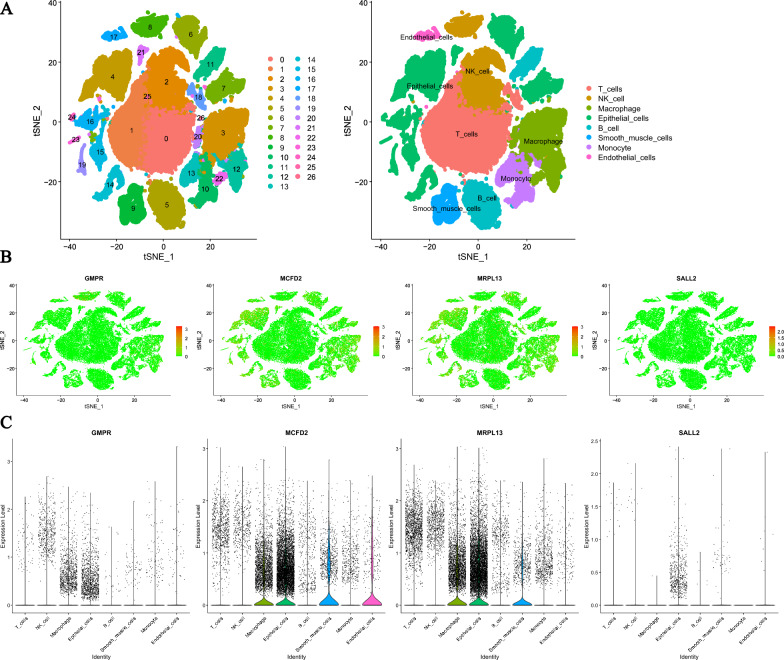
Verification of OCIRGs by sc-RNA seq. **A** tSNE plot of cells generated from LUAD tissues. The images are color-coded by cell clusters, with the cells clustering into 27 subclusters. Each point represents a LUAD cell. **B** Expression of ODFRGs visualized in tSNE in LUAD. **C** Violin plot depicting the expression of ODFRGs in LUAD clusters

This study used 12 samples for single-cell sequencing, considering factors such as RNA content, gene expression quantity, RNA integrity, cell category, and reaction quality control when selecting cells. A total of 57,219 cells were obtained. Through t-SNE cluster analysis, these cells were divided into 27 cell clusters, and annotation tools were used for classification, including T cells, B cells, NK cells, macrophages, monocytes, epithelial cells, smooth muscle cells, and endothelial cells (Fig. 7A). In order to further analyze the expression differences of ODFRGs in different cell types, we used violin plots and t-SNE plots for visualization analysis. The results showed that the expression levels of MCFD2 and MRPL13 in macrophages were higher than in other cell types (Fig. 7B, C).

### Analysis of prognostic model-related signaling pathways and functions

**Fig. 8:**
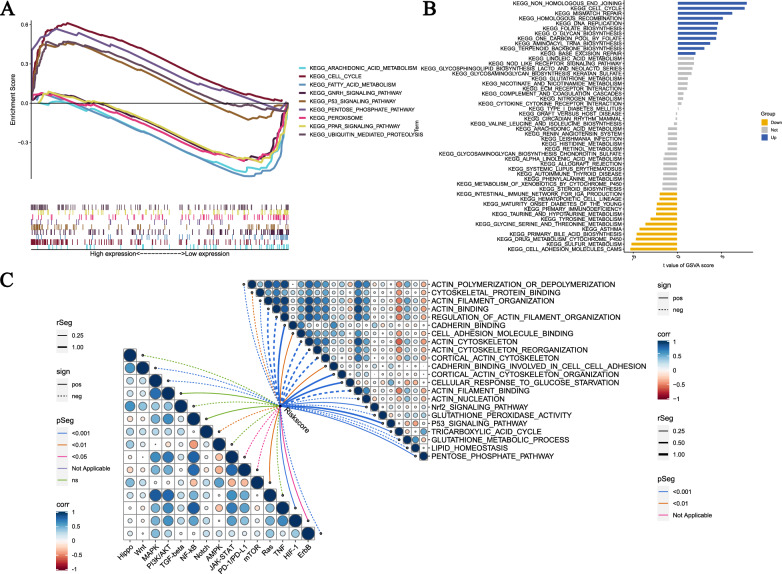
Biological functions. **A** Significantly enriched pathways in the high-risk and low-risk groups. The extremum located on the left side indicates a positive association between risk scores and pathway activity, and vice versa. **B** There is a significant difference in pathways between high-risk and low-risk groups. Blue bars represent a positive correlation between risk scores and pathway activity, while yellow bars indicate the opposite. **C** The correlation between riskscore and tumor important pathways, as well as disulfidptosis/ferroptosis-related functions

According to the biological analysis using GSEA software, the pathways Cell cycle, phosphate pathway, peroxisome, ubiquitin mediated proteolysis, and p53 signaling pathway are active in the high-risk group, while the pathways Arachidonic acid metabolism, fatty acid metabolism, GnRH signaling pathway, pentose phosphate pathway, and PPAR signaling pathway are active in the low-risk group (Fig. 8A). The results of GSVA enrichment analysis show that the functions DNA replication, homologous recombination, mismatch repair, and cell cycle are enriched in the high-risk group, while the functions Sulfur metabolism, glycine serine and threonine metabolism, tyrosine metabolism, primary immunodeficiency, and intestinal immune network for IgA production are enriched in the low-risk group (Fig. 8B). In addition, important tumor pathways, such as Wnt, MAPK, Notch, JAK-STAT, PD-1/PD-L1, mTOR, TNF, HIF-1, and ErbB, are all related to the risk score, and pathways and functions related to disulfidptosis and ferroptosis are also related to the risk score (Fig. 8C).

### Analysis of the mutation landscape

**Fig. 9:**
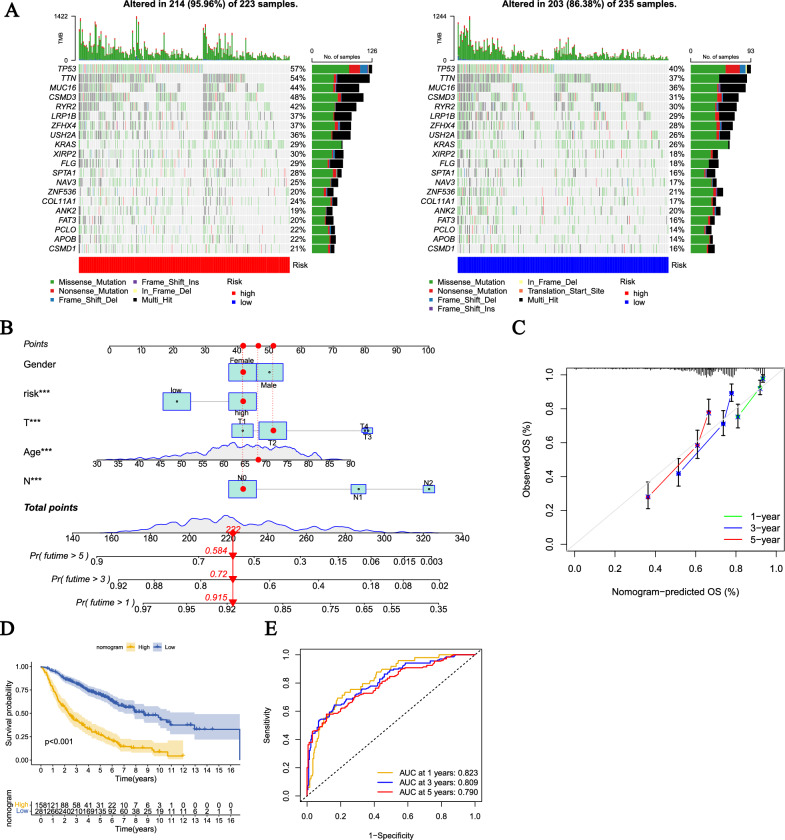
Mutational landscape differential analysis of risk subtypes and construction of a nomogram. **A** Overview of the top 20 mutated genes in high-risk and low-risk populations. The bars above show the total amount of gene mutations and the corresponding mutation types. The right column displays the mutation frequency of the top 20 mutated genes. **B** Nomogram for 1-, 3-, and 5-year overall survival prediction. The red line shows an example of predicting the prognosis. **C** Calibration plots for agreement tests between predicted and actual OS. **D** KM analysis was used to compare the differences in survival between high and low nomogram score groups. **E** ROC analysis was used to evaluate the ability and accuracy of the nomogram

Gene mutations are of great significance to tumors because they are one of the main causes that lead to the formation and development of tumors. Gene mutations can change the gene expression and protein structure in cells, leading to abnormal growth and division of cells, and eventually forming tumors. Analysis of the mutation status of high-risk and low-risk groups shows that the mutation frequency of high-risk group genes such as TP53, TTN, MUC16, CSMD3, RYR2, LRP1B, ZFHX4, and USH2A are higher than that of low-risk group (Fig. 9A).

### Construction of nomogram

In order to further utilize the DFRG prognosis model for the prediction of LUAD patient survival, we additionally included clinical data in the construction of the nomogram, including Gender, T stage, N stage, and Age (Fig. 9B). The red line in the figure indicates example data. In addition, we presented the calibration curve of the nomogram (Fig. 9C). To verify the model, we analyzed the survival situation of the high and low scoring groups using the KM curve. The results showed that the OS of the high-score group was significantly lower than that of the low-score group (Fig. 9D). The ROC curve results for 1 year, 3 years, and 5 years were AUC values of 0.823, 0.809, and 0.790, respectively (Fig. 9E).

### Verification of ODFRGS expression

**Fig. 10:**
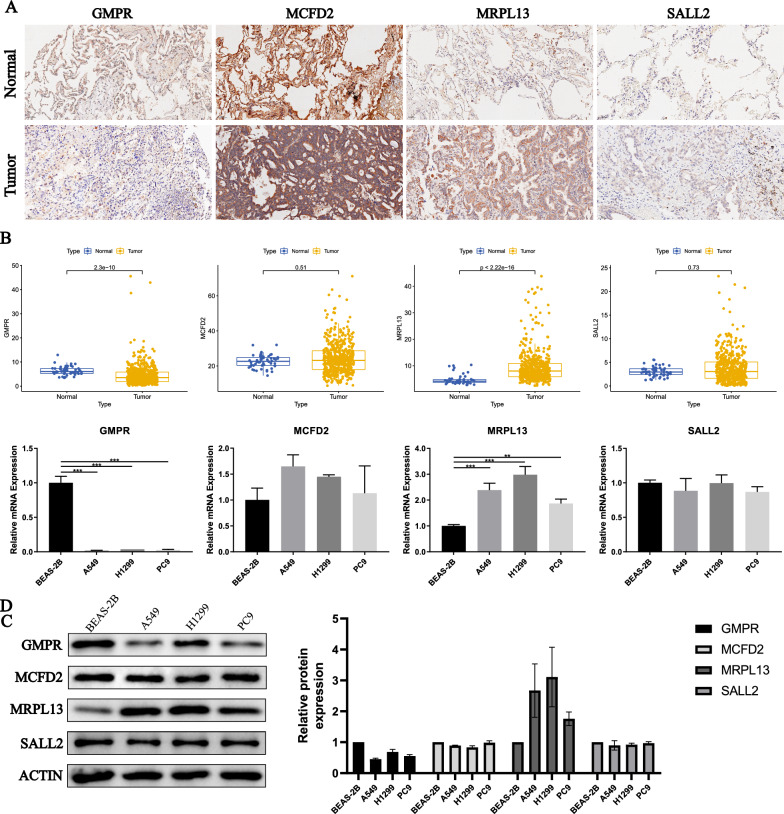
Analysis of ODFRGs expression. **A** IHC verification of the expression level of ODFRGs in the LUAD tissue and surrounding tissue. **B** Analysis of the expression levels of ODFRGs based on RNA-seq data between normal and tumor samples. **C** PCR verification ODFRGS's expression level. **D** Western Bloting verifies the expression of ODFRGS in 1 normal cell strain and three types of LC cells

To verify the expression levels of ODFRG in normal and LUAD samples, we analyzed the TCGA LUAD cohort. The results showed that, compared with normal samples, GMPR was under-expressed in tumors, MRPL13 was overexpressed in lung adenocarcinoma, while the expression of MCFD2 and SALL2 between normal lung tissue and lung adenocarcinoma tissue was not different (Fig. 10A). We subsequently demonstrated the immunohistochemical results of ODFRGs in LUAD tissue (Fig. 10B). In addition, qPCR (Fig. 10C) and western blotting (Fig. 10D) also get got the same result.

### In vitro functional assessment of GMPR

**Fig. 11:**
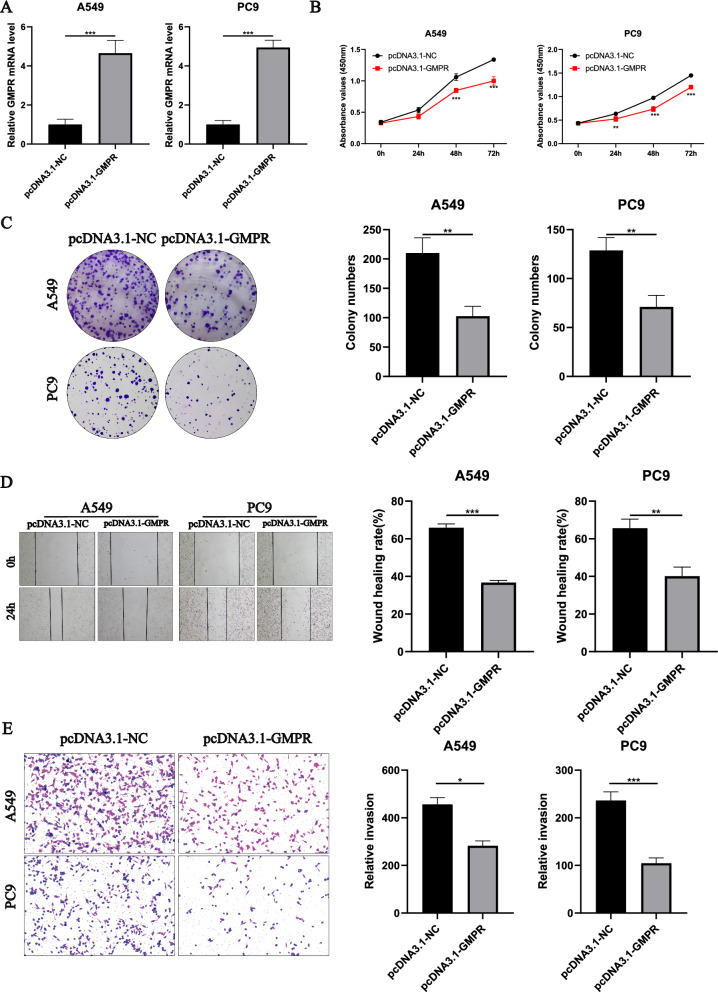
Validates the role of the key gene GMPR in lung cancer cell lines in vitro. **A** The overexpression of GMPR significantly enhanced its expression in the A549 and PC9 cell lines (**P < 0.001). **B** After the overexpression of GMPR in A549 and PC9 cell lines, the activity of lung adenocarcinoma cells significantly decreased (**P < 0.01, ***P < 0.001). **C** Clone formation assay results showed that the ability of colony formation in A549 and PC9 cell lines was significantly reduced after GMPR overexpression (**P < 0.01). **D** The control group demonstrated a stronger migration ability than the experimental group in the wound healing experiment in A549 and PC9 cell lines (**P < 0.01, ***P < 0.001). **E** Overexpression of GMPR attenuated the invasive ability of A549 and PC9 cell lines (*P < 0.05, ***P < 0.001)

In A549 and PC9 cell lines, the expression levels of GMPR were significantly increased after overexpression of GMPR (Fig. 11A; ***P < 0.001). Furthermore, overexpression of GMPR significantly reduces the proliferation of lung adenocarcinoma cells in A549 and PC9 cell lines. (Fig. 11B; **P < 0.01). Subsequently, colony formation analysis showed that the ability of colony formation in A549 and PC9 cell lines was significantly decreased after overexpression of GMPR (Fig. 11C; **P < 0.01). Overexpression of GMPR significantly reduced the migration ability of A549 and PC9 cell lines in wound healing experiments (Fig. 11D; P < 0.01, P < 0.001). In the transwell assay, overexpression of GMPR significantly decreased the invasive ability of A549 and PC9 cell lines (Fig. 11E; *P < 0.05, ***P < 0.001).

## Discussion

In recent decades, significant progress has been made in the treatment of lung cancer. Emerging technologies such as targeted therapy, immunotherapy, and multidisciplinary treatment models have been applied in clinical practice, and research results from clinical trials have provided more effective treatment options [16]. However, we still face many challenges to control the development of lung cancer and reduce the mortality rate of patients. In addition, we still do not fully understand the etiology and pathogenesis of lung cancer. Therefore, in the research process, exploring new molecular markers and cell signaling pathways, analyzing in-depth the molecular mechanisms associated with lung cancer, will provide important theoretical guidance for new oral chemotherapy drugs and immunotherapy [17].

In February 2023, a team of professors from the MD Anderson Cancer Center at the University of Texas, including Professor Gan Boyi and Professor Chen Junjie, first proposed a cell death process called disulfidptosis in the journal Nature Cell Biology [3]. This type of cell death occurs more in cells with high expression of SLC7A11 and abnormal accumulation of disulfide compounds caused by extreme glucose starvation leads to death. The characteristic manifestations of this death process are abnormal formation of disulfide bonds in muscle actin cytoskeleton proteins and collapse of F-actin. Lack of glucose affects the generation of NADPH in the pentose phosphate pathway, and the main function of SLC7A11 is to uptake cysteine [3]. When glucose starvation occurs, the accumulation of cysteine in the cells will cause the generation of disulfide stress, leading to an imbalance of the redox state and the occurrence of disulfidptosis. During this process, there are significant changes in cell morphology, including cell contraction and F-actin contraction, and the morphology of muscle actin cytoskeleton undergoes changes, including corresponding changes in disulfide bonds [18]. Ferroptosis is an important type of cell death, which mainly occurs due to the combined action of oxidative stress, membrane lipid peroxidation, and other factors inside the cell, resulting in cell damage and death [19]. Compared with other cell apoptosis and necrosis pathways, ferroptosis is characterized by lipid peroxidation and has multiple specific regulation mechanisms [20].

Ferroptosis plays a significant role in lung adenocarcinoma. Studies have shown that ferroptosis is involved in the development and treatment of lung adenocarcinoma, providing a new theoretical basis for its treatment. Lung adenocarcinoma cells have varying sensitivities to ferroptosis-inducing drugs [11]. For example, overexpression of the protein NFS1 in lung adenocarcinoma cells can promote tumor growth and reduce sensitivity to ferroptosis drugs. Inhibiting NFS1 leads to cellular iron depletion and increased sensitivity to ferroptosis [21]. Additionally, a protein called STYK1 has been identified in lung adenocarcinoma cells, which promotes lung cancer metastasis and suppresses ferroptosis through upregulating GPX4 expression [22]. Another protein, called LSH, a chromatin remodeling protein, inhibits ferroptosis by activating genes associated with metabolism [23]. Understanding the value of ferroptosis in lung adenocarcinoma is crucial for its treatment. Firstly, ferroptosis is a unique form of cell death that differs from traditional apoptosis. This means that regulating ferroptosis opens up a new treatment pathway and overcomes limitations of traditional therapies. Secondly, specific proteins in lung adenocarcinoma cells, such as NFS1, STYK1, and LSH, play important regulatory roles in the process of ferroptosis. Understanding and intervening in these regulatory factors can enhance the tumor's sensitivity to ferroptosis drugs, thereby improving the therapeutic effect of lung adenocarcinoma. Additionally, identifying drugs and compounds that induce ferroptosis contributes to the development of new treatment strategies[24]. In conclusion, the study of ferroptosis in lung adenocarcinoma provides a new theoretical basis for its treatment. By gaining a deep understanding of the regulatory mechanisms and influencing factors of ferroptosis, we can develop more targeted treatment methods to enhance the effectiveness of lung adenocarcinoma treatment. Future research should further explore the specific mechanisms of ferroptosis in lung cancer and develop safer and more effective ferroptosis inducers for better clinical applications. As for disulfidptosis, there is limited research at present. It is known that disulfidptosis plays an important role in renal cancer. Studies have shown that the expression level of SLC7A11 in tumor tissues of renal cancer patients is significantly elevated, which is closely associated with tumor growth and progression. When renal cancer cells are exposed to a glucose-deprived environment, SLC7A11-induced disulfidptosis occurs, leading to cell death. During the process of disulfidptosis, the high expression of SLC7A11 induces cell death by reducing the level of NADPH and causing the accumulation of reactive oxygen species [25]. In fact, utilizing the mechanism of disulfidptosis can be a potential strategy for treating renal cancer. One approach is to target SLC7A11-overexpressing renal cancer cells and inhibit the glucose transport pathway to induce disulfidptosis and suppress tumor growth [3]. Additionally, exploring other proteins and signaling pathways that regulate disulfidptosis could promote cell death in renal cancer cells. Therefore, the discovery of disulfidptosis provides new ideas and strategies for the treatment of renal cancer and offers inspiration for the treatment of other diseases. However, due to the relatively short period of time that disulfidaptosis, a cell death process, was discovered, there is currently limited research on lung cancer. Currently, there are some bioinformatics studies related to lung cancer, but the exploration of the mechanism of disulfidaptosis in lung cancer still has gaps and requires further investigation by future researchers [26–31].

This study discovered the connection between disulfidptosis and ferroptosis. Professors Gan and Chen pointed out in their article the important role of SLC7A11 in disulfidptosis [3]. Regardless of the deformation of the cytoskeleton, disulfide bond generation, F-actin contraction, or the occurrence of disulfidptosis, SLC7A11 played a mediating role. Previous research has shown that SLC7A11 plays a crucial role in ferroptosis [17, 32]. The function of SLC7A11 is to promote the entry of cysteine (Cys) into cells and increase the synthesis of glutathione (GSH). Glutathione is a very important antioxidant that can effectively remove excess ROS in cells [33]. Therefore, when the expression level of SLC7A11 is reduced, its function is inhibited, or there is a mutation, the amount of GSH synthesized by cells will decrease, which in turn decreases GPX4 activity, resulting in ROS accumulation and cell death. Based on this knowledge, it is believed that SLC7A11 is one of the key regulatory factors in the process of controlling ferroptosis, and its mechanism of action needs to be paid special attention to.

This study screened and analyzed four ODFRGs, namely GMPR, MCFD2, MRPL13, and SALL2. We further analyzed the prognostic model constructed by ODFRGs, including the tumor immune microenvironment and enriched functional pathways in the risk subtype, as well as the analysis of ODFRGs using single-cell sequencing.

GMPR(Guanosine Monophosphate Reductase) is an enzyme that converts guanosine monophosphate (GMP) to inosine monophosphate (IMP) and plays a role in purine nucleotide metabolism pathways. Multiple studies have shown that [34, 35] GMPR plays an important biological role in melanoma. The expression of this enzyme can inhibit the invasion and metastasis of melanoma cells, thereby reducing the malignancy of melanoma. In addition, maintaining an appropriate level of GMPR expression potential can inhibit the development of melanoma, making GMPR a potential target for the treatment of melanoma. In our study, GMPR was found to be downregulated in lung adenocarcinoma and was correlated with poor prognosis. The expression of GMPR was positively correlated with the infiltration abundance of resting mast cells.

MCFD2 (Multiple Coagulation Factor Deficiency 2) is a protein related to coagulation factors. Recent studies have shown that [36] MCFD2 is involved in the development of oral cancer, and its expression level in oral cancer cells is significantly higher than in normal cells. Studies have also found that reducing the expression of MCFD2 can effectively inhibit the growth and metastasis of oral cancer. The database results show that high expression of MCFD2 in LUAD is associated with poor prognosis. The expression of MCFD2 is positively correlated with the abundance of M2 macrophage infiltration and negatively correlated with the abundance of memory B cells infiltration.

MRPL13(Mitochondrial Ribosomal Protein L13) is one of the components of mitochondrial ribosomes and has multiple oncogenic mechanisms. In breast cancer, MRPL13 is involved in cell proliferation and EMT process, and is associated with poor prognosis of patients [37–39]. In non-small cell lung cancer, the expression of MRPL13 is also higher than that of normal tissues or cells, which can promote the proliferation of tumor cells and induce apoptosis [40]. Studies have shown that inhibiting MRPL13 can slow down the proliferation of breast cancer cells and EMT process, while reducing the resistance of non-small cell lung cancer cells to anthracyclines chemotherapy drugs. Currently, there is no evidence to prove the important role of MRPL13 in immune cell regulation in the tumor microenvironment, thus further research is needed to clarify its role in tumor treatment. Overall, MRPL13 is one of the important directions of tumor treatment research. This study found that MRPL13 is highly expressed in lung adenocarcinoma and is associated with poor prognosis. Immune infiltration analysis showed that the expression level of MRPL13 is highly correlated with the infiltration level of activated memory CD4 T cells.

MRPL13 (Mitochondrial Ribosomal Protein L13) is one of the components of mitochondrial ribosomes and has various carcinogenic mechanisms. In breast cancer, MRPL13 is involved in cell proliferation and EMT processes and is associated with poor prognosis in patients [37–39]. In non-small cell lung cancer, the expression of MRPL13 is also higher than that in normal tissues or cells, promoting tumor cell proliferation and inducing cell apoptosis [40]. Studies have shown that inhibiting MRPL13 can slow down the proliferation and EMT process of breast cancer cells [37]. Overall, MRPL13 is one of the important directions in tumor treatment research. This study found that MRPL13 is highly expressed in lung adenocarcinoma and is associated with poor prognosis. Immunoinfiltration analysis shows that the expression level of MRPL13 is highly correlated with the infiltration level of activated memory CD4 + T cells.

SALL2 (Spalt Like Transcription Factor 2) is a transcription factor belonging to the Spalt-like family. In ovarian cancer, SALL2 is believed to have a role in inhibiting tumor development [41], but it may promote tumor growth in glioblastoma [42]. Studies have shown that SALL2 can inhibit cell proliferation by regulating the transition from G1 to S phase and suppressing the expression of Cyclin D1 and E1. SALL2 plays an important role in breast cancer. SALL2 downregulation leads to loss of sensitivity to hormone therapy in breast cancer patients, affecting cell proliferation and apoptosis [39]. SALL2 directly regulates the transcription levels of ERα and PTEN genes, which have a specific impact on the treatment and prognosis of breast cancer patients.This regulatory effect can help prevent the occurrence of cancer. In addition, SALL2 also induces cell cycle arrest and apoptosis, which further contributes to the suppression of tumor development. In this study, it was found that SALL2 has a protective effect in LUAD. The higher the expression level of SALL2, the earlier the clinical staging of LUAD patients.

The results of single-cell sequencing revealed a significant upregulation of MCFD2 and MRPL13 expression in macrophages, vital cellular components in tumor microenvironments. Macrophages demonstrate diverse phenotypes, with M1 macrophages exhibiting anti-tumor activity and M2 macrophages promoting tumor growth. Tumor-associated macrophages (TAMs) play a pivotal role in photodynamic therapy (PDT), a treatment modality relying on the ability of macrophages to uptake systemically administered photosensitizers. Consequently, macrophage activity largely contributes to the localization and local fluorescence of photosensitizers within tumors. Upon PDT treatment, macrophages enriched with photosensitizers (predominantly M2 macrophages) sustain considerable damage and become replaced by newly recruited M1 macrophages. These macrophages play a critical role in the efficacy of PDT by facilitating the clearance of apoptotic cancer cells and processing/presenting tumor antigens to T lymphocytes. As a result, macrophages hold substantial significance in both immunotherapy and PDT for tumor management. Additionally, the heightened expression of MCFD2 and MRPL13 within macrophages suggests their potential involvement in macrophage-related functions.

The proportional hazards regression model in this study was also constructed based on the aforementioned four ODFRGs. The functions associated with the model include Arachidonic acid metabolism, cell cycle, peroxisome, and ubiquitin mediated proteolysis.

Enzymes related to Arachidonic acid metabolism [43], such as cyclooxygenase and lipoxygenase, play a critical role in the development and progression of cancer. These enzymes' metabolites include prostaglandin E2, leukotriene A4, and thromboxane A2, which have been shown to stimulate cell proliferation, angiogenesis, and cancer cell metastasis. Therefore, research on inhibiting these enzymes has become a hot topic in cancer treatment. Many natural products, such as bioactive substances and dietary plant compounds, are believed to be potential therapeutic drugs.

Cell cycle [44] refers to the process by which cells grow and divide, including the G1, S, G2, and M phases. The regulation of the cell cycle mainly depends on activators and inhibitors. Cells will perform different life cycle checkpoints at different stages to ensure cell health and prepare for division. Errors in the cell cycle may lead to cell apoptosis or genetic instability.

Peroxisome [45] plays an important metabolic role in tumor cells, especially in regulating the oxidation–reduction state and improving fatty acid oxidation and ether phospholipid synthesis. Therefore, studying the function of Peroxisome in tumor metabolism can provide new potential targets for cancer treatment (Additional file 3).

Ubiquitin mediated proteolysis[46] plays a crucial role in cancer. E3 ubiquitin ligases such as APC/C (ANAPHASE-PROMOTING COMPLEX/CYCLOsome) and SCF (SKP1-CUL1-F-box protein complex) regulate the ubiquitin signaling pathway to ensure the progression of the cell cycle. Correct ubiquitination process can prevent uncontrolled cell proliferation and tumor occurrence. Therefore, the treatment strategy targeting E3 ubiquitin ligases has become one of the novel approaches to cancer treatment (Additional file 4).

## Conclusion

Through the analysis of public database data, this study identified prognostic genes in LUAD and ultimately selected 4 ODFRGs to construct a prognostic model. The model can accurately predict the prognosis and level of immune infiltration among LUAD patients, providing a reliable basis for clinicians to make personalized treatment decisions. Moreover, the elevated expression of GMPR in ODFRGs in lung adenocarcinoma reduces the proliferation, migration, and invasion of lung cancer cells. Additionally, the results of this research provide important foundational data for further exploring LUAD and tumor microenvironment.

### Supplementary Information

**Additional file 1: Figure S1.** (**A, B**) Unsupervised clustering of GSE68465 based on disulfidptosis-related genes and ferroptosis-related genes showed the best clustering effect when K=2. (**C**) KM curve demonstrated differences in overall survival (OS) between the two clusters related to disulfidptosis. (**D**) Univariate Cox analysis was performed on DFRGs, and survival-related DFRGs were selected. (**E, F**) Univariate Cox and multivariate Cox analyses were conducted on clinical features and riskscore, revealing independent prognostic factors.

**Additional file 2. ** Table S1.

**Additional file 3. **Vendors and catalog numbers of the antibodies.

**Additional file 4. **The primer names and corresponding primer sequences.

## Data Availability

Data for this study were obtained from public databases.
