# Supplementary material for: Leveraging a disulfidptosis/ferroptosis-based signature to predict the prognosis of lung adenocarcinoma
Source: Cancer Cell Int. 2023 Nov 9;23:267. doi: 10.1186/s12935-023-03125-z (PMC10634118; doi:10.1186/s12935-023-03125-z)
Supplement: Supplementary file 1 — Additional file 1: Figure S1. (A, B) Unsupervised clustering of GSE68465 based on disulfidptosis-related genes and ferroptosis-related genes showed the best clustering effect when K=2. (C) KM curve demonstrated differences in overall survival (OS) between the two clusters related to disulfidptosis. (D) Univariate Cox analysis was performed on DFRGs, and survival-related DFRGs were selected. (E, F) Univariate Cox and multivariate Cox analyses were conducted on clinical features and riskscore, revealing independent prognostic factors. [file 12935_2023_3125_MOESM1_ESM.pdf]

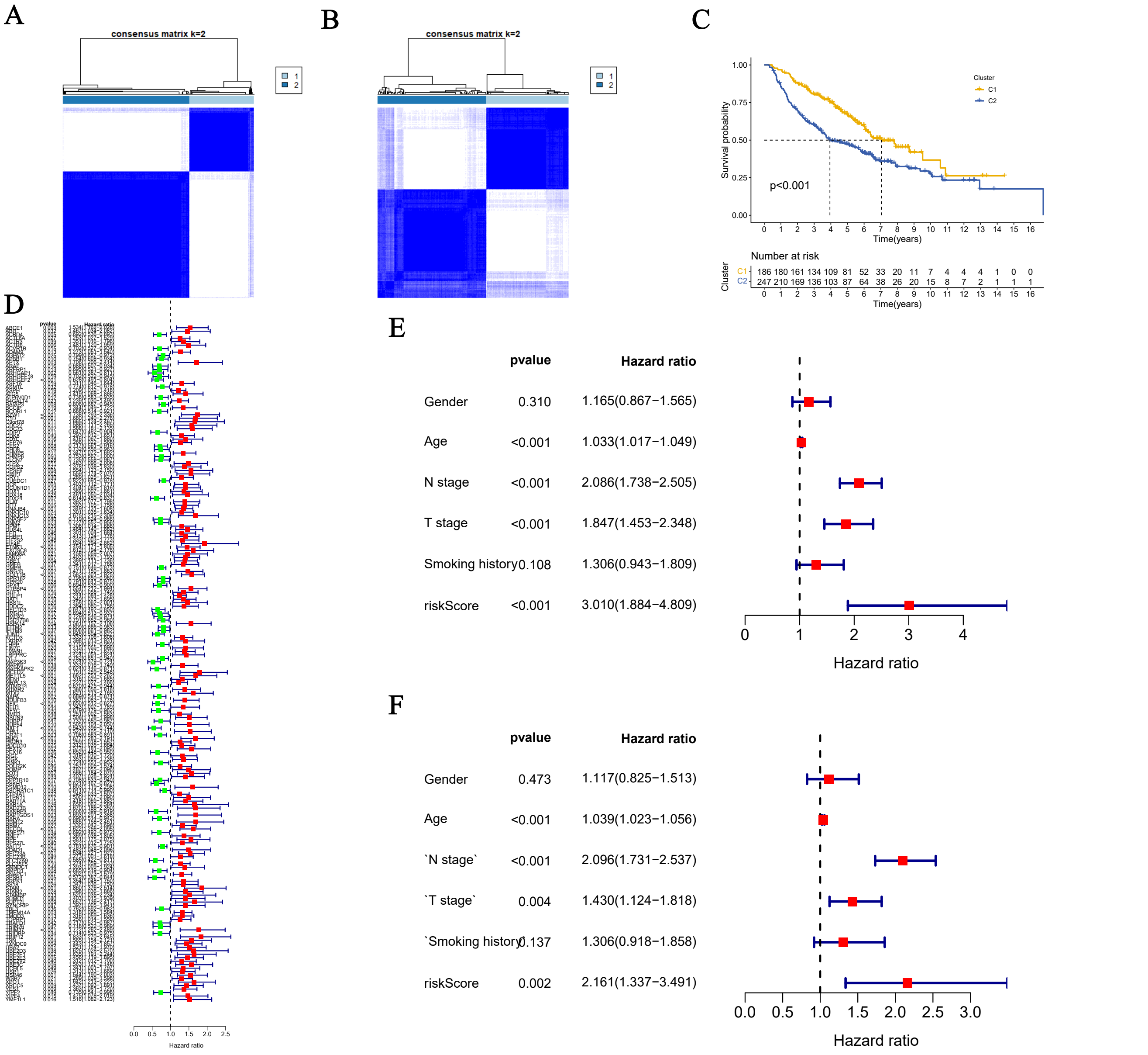
A
consensus matrix k=2
1
2
B
consensus matrix k=2
1
2
C
Survival probability
1.00
0.75
0.50
0.25
0.00
Cluster
C1
C2
p<0.001
0 1 2 3 4 5 6 7 8 9 10 11 12 13 14 15 16
Time(years)
Number at risk
Cluster
C1 186 180 161 134 109 81 52 33 20 11 7 4 4 1 0 0
C2 247 210 169 136 103 87 64 38 26 20 15 8 7 2 1 1 1
0 1 2 3 4 5 6 7 8 9 10 11 12 13 14 15 16
Time(years)
D
pvalue
Hazard ratio
0.0 0.5 1.0 1.5 2.0 2.5
Hazard ratio
E
pvalue
Hazard ratio
Gender 0.310 1.165(0.867−1.565)
Age <0.001 1.033(1.017−1.049)
N stage <0.001 2.086(1.738−2.505)
T stage <0.001 1.847(1.453−2.348)
Smoking history 0.108 1.306(0.943−1.809)
riskScore <0.001 3.010(1.884−4.809)
0 1 2 3 4
Hazard ratio
F
pvalue
Hazard ratio
Gender 0.473 1.117(0.825−1.513)
Age <0.001 1.039(1.023−1.056)
`N stage` <0.001 2.096(1.731−2.537)
`T stage` 0.004 1.430(1.124−1.818)
`Smoking history` 0.137 1.306(0.918−1.858)
riskScore 0.002 2.161(1.337−3.491)
0.0 0.5 1.0 1.5 2.0 2.5 3.0
Hazard ratio
