# Supplementary material for: Leveraging a disulfidptosis/ferroptosis-based signature to predict the prognosis of lung adenocarcinoma
Source: Cancer Cell Int. 2023 Nov 9;23:267. doi: 10.1186/s12935-023-03125-z (PMC10634118; doi:10.1186/s12935-023-03125-z)
Supplement: Supplementary file 2 — Additional file 2. Table S1 [file 12935_2023_3125_MOESM2_ESM.pdf]

	Decision Tree	LASSO	Random Forest	GBDT	XGBoost	AVG	
STAM	0	0.193307	0.021615	0.022395	0.015784	0.05062	(1)
MCFD2	0.012671	0.137993	0.016101	0.060207	0.006438	0.046682	(2)
GMPR	0.011103	0.114598	0.000907	0.011962	0.014171	0.030548	(3)
MRPL13	0.027238	0	0.036498	0.072193	0.009307	0.029047	(4)
ARHGEF2	0.002588	0.073973	0.002536	0.012569	0.004727	0.019278	(5)
APTX	0.010659	0.065506	0.007576	0	0.011613	0.019071	(6)
SALL2	0.012637	0.050212	0.006	0.019072	0.004313	0.018447	(7)
TRIM37	0.01645	0	0.01749	0.040072	0.01764	0.01833	(8)
OR2F1	0	0.062986	0.004299	0.01598	0.002444	0.017142	(9)
HECTD3	0.007159	0.053388	0.00445	0.005475	0.011062	0.016307	(10)
